# Clinically Explainable Prediction of Immunotherapy Response Integrating Radiomics and Clinico-Pathological Information in Non-Small Cell Lung Cancer †

**DOI:** 10.3390/cancers17162679

**Published:** 2025-08-18

**Authors:** Jhimli Mitra, Soumya Ghose, Rajat Thawani

**Affiliations:** 1 GE HealthCare, Niskayuna, NY 12309, USA; soumya.ghose@gehealthcare.com; 2 Division of Hematology and Oncology, Knight Cancer Institute, Oregon Health & Science University (OHSU), Portland, OR 97239, USA; thawani@ohsu.edu

**Keywords:** non-small cell lung cancer, immunotherapy response, radiomics, multimodal analysis, machine learning, large-language model, clinical explanation

## Abstract

Background/Objectives: Immunotherapy is a viable therapeutic approach for non-small cell lung cancer (NSCLC). Despite the significant survival benefit of immune checkpoint inhibitors PD-1/PD-L1, on average; the objective response rate is around 20% as monotherapy and around 50% in combination with chemotherapy. While PD-L1 IHC is used as a predictive biomarker, its accuracy is subpar. Methods: In this work, we develop a machine learning (ML) method to predict response to immunotherapy in NSCLC from multimodal clinicopathological biomarkers, tumor and peritumoral radiomic biomarkers from CT images. We further learn a graph structure to understand the associations between biomarkers and treatment response. The graph is then used to create sentences with clinical hypotheses that are finally used in a Large Language Model (LLM) that explains the treatment response predicated on the biomarkers that are comprehensible to clinicians. From a retrospective study, a training dataset of NSCLC with n = 248 tumors from 140 subjects was used for feature selection, ML model training, learning the graph structure, and fine-tuning LLM. Results: An AUC = 0.83 was achieved for prediction of treatment response on a separate test dataset of n = 84 tumors from 47 subjects. Conclusions: Our study therefore not only improves the prediction of immunotherapy response in patients with NSCLC from multimodal data but also assists the clinicians in making clinically interpretable predictions by providing language-based explanations.

## Simple Summary

Only 20% patients with non-small cell lung cancer respond to immunotherapy alone and 40% to immunotherapy in combination with chemotherapy. The PD-L1 value cutoff from immunohistochemistry that is used to select patients who would respond to immunotherapy lacks accuracy. A combination of other clinical biomarkers and radiomic biomarkers from CT should be analyzed for the selection of patients who would benefit from immunotherapy. The aim of our retrospective study was to develop a machine learning model that predicted treatment response from multimodal data (clinical, peritumoral and tumoral radiomics features). This combination of features outperfomed using radiomics or clinical variables alone. A large language model was further used to explain the response predictions in natural-language using the biomarkers that are comprehensible to clinicians.

## 1. Introduction

Immune checkpoint inhibitors have transformed the landscape of cancer treatment, especially in the non-small cell lung cancer (NSCLC) [1,2,3]. There are FDA approvals range in the metastatic setting, but also in the neoadjuvant, adjuvant or consolidative setting after chemoradiation. Currently, the use is appropriate in all patients without oncogene alterations where tumors are immune-resistant. While this indication is broad, the response rate to immune checkpoint inhibitors varies from 10–60% dependent on multiple factors like biomarkers, or combination partners. There is a significant need for the development of novel biomarkers of response to immunotherapy to better characterize patients who might require escalation or de-escalation of their cancer therapies.

Currently there are only two predictive biomarkers for immunotherapy in NSCLC by the US FDA, i.e., tumor PD-L1 expression assessed by immunohistochemistry (IHC) [4] and tumor mutation burden (TMB) [5,6,7]. Although programmed death-ligand 1 (PD-L1) protein expression by immunohistochemistry (IHC) has played a role as the principal predictive biomarker for immunotherapy; PD-L1 ≥50% being a strong predictor, up to 50–60% of these patients still do not respond, suggesting additional resistance mechanisms. The cut-offs of high PD-L1 being 50% is also arbitrary and the levels can vary within different tumor regions (spatial heterogeneity) and change over time with dynamic changes due to response to therapy, inflammation, or natural history of cancer (temporal heterogeneity). Moreover, PD-L1 lacks optimal performance with issues in different approved companion diagnostic assays [8].

TMB thresholds, such as the commonly cited 10 mut/Mb, are somewhat arbitrary and can vary depending on the testing method [9]. Moreover, high TMB does not consistently predict immune infiltration or response to immunotherapy, as seen in Microsatellite Stable (MSS) colorectal cancer. These limitations underscore the complexity of anti-tumor immune responses, which involve multiple cell types, signaling networks, and interactions with the tumor microenvironment—making it difficult to isolate genomic predictors of response.

To address these limitations, imaging-based biomarkers offer a promising alternative. Computed tomography (CT), the most widely used modality for lung cancer diagnosis, treatment planning, and monitoring, provides an accessible platform for integrating radiomics into clinical practice. CT radiomics from pre-treatment scans can quantitatively capture subtle tumor characteristics and have been linked to prognosis, recurrence, and survival in NSCLC [10,11,12,13,14]. Recently, attention has turned to peritumoral radiomics—features extracted from tissue surrounding the tumor—as potential markers of the immune micro-environment [15,16,17,18]. While the tumor core frequently exhibits necrosis, hypoxia, or dense fibrosis—conditions that can limit immune infiltration—the peritumoral region is where immune-tumor interactions are most active. This zone contains critical components such as T-cells, macrophages, fibroblasts, and vasculature, making it a hotspot for immune signaling. Biologically, this peripheral region correlates strongly with the presence of CD8+ T-cell infiltration and immune gene expression signatures, both are known predictors of ICI response [19,20]. However, variability in imaging protocols and radiomic feature extraction methods remains a challenge, underscoring the need for further validation in this area [16].

Based on the previous studies [21,22,23,24], we hypothesize that multimodal data analysis can provide a more comprehensive picture of the disease by combining information from various sources like medical imaging, genetic analysis, and clinical data, leading to a more accurate prediction of response to immunotherapy [25]. While multimodal analysis was attempted on multicenter cohorts for predicting treatment response [26,27], a rigorously curated dataset from routinely collected clinical care data was made publicly available in [28], and a multimodal analysis that included, CT, histology, genomics and clinical data was performed. Although several subsets of multimodal data were used in different studies, the tumoral and peritumoral radiomics have not been analyzed with clinical variables in previous studies. Aiming to explore these, we developed a multimodal machine learning model using Random Forest (RF). Further to the prediction model, we also learned a Bayesian graph defining the associations between clinical variables and treatment outcome. This graph is used to establish apriori knowledge to fine-tune a Large Language Model (LLM) that is further used to explain the treatment response in clinically interpretable language.

## 2. Materials and Methods

### 2.1. Study Cohort

This study used a publicly available, retrospective subset of data from n = 187 patients of the 247 patients with NSCLC from the Memorial Sloan Kettering Cancer Center (MSKCC) cohort (https://www.synapse.org/#!Synapse:syn26642505, accessed on 12 August 2025) [28]. These 187 (76%) patients had disease that was clearly separable from the adjacent organs and that had CT images, PD-L1 expression scores, Tumor Metabolic Burden (TMB) and clinical data such as smoking status and age. These patients included 169 (90%) with lung parenchymal lesions, 20 (11%) with pleural lesions and 67 (36%) with pathologically enlarged lymph nodes, typically with more than one-type of lesions present in one patient. The treatment responders vs. non-responders were binarized. The training and test cohorts from these 187 patients were separated using a stratified K-fold (4-fold) method, that split the dataset into four equal-sized folds. We considered one fold (n = 47 patients) as the test cohort and other 3-folds comprising the training cohort (n = 140 patients). The stratified K-fold is a helpful data-splitting strategy for datasets with imbalanced classes (136 non-responders vs. 51 responders). It ensures that each fold in the K-fold process maintains the same proportion of classes as the original dataset. This helps in creating more reliable and less biased model performance estimates. Feature selection through cross-validation techniques, training the ML model, Bayesian modeling, creating Retrieval Augmented Generation (RAG) contexts have been performed only with the training cohort, while a separate test cohort was used for ML model, Bayesian model predictions and explaining the response predictions using LLM. Table 1 shows the characteristics of the patients in the training and test cohorts.

### 2.2. Study Design

**Figure 1:**
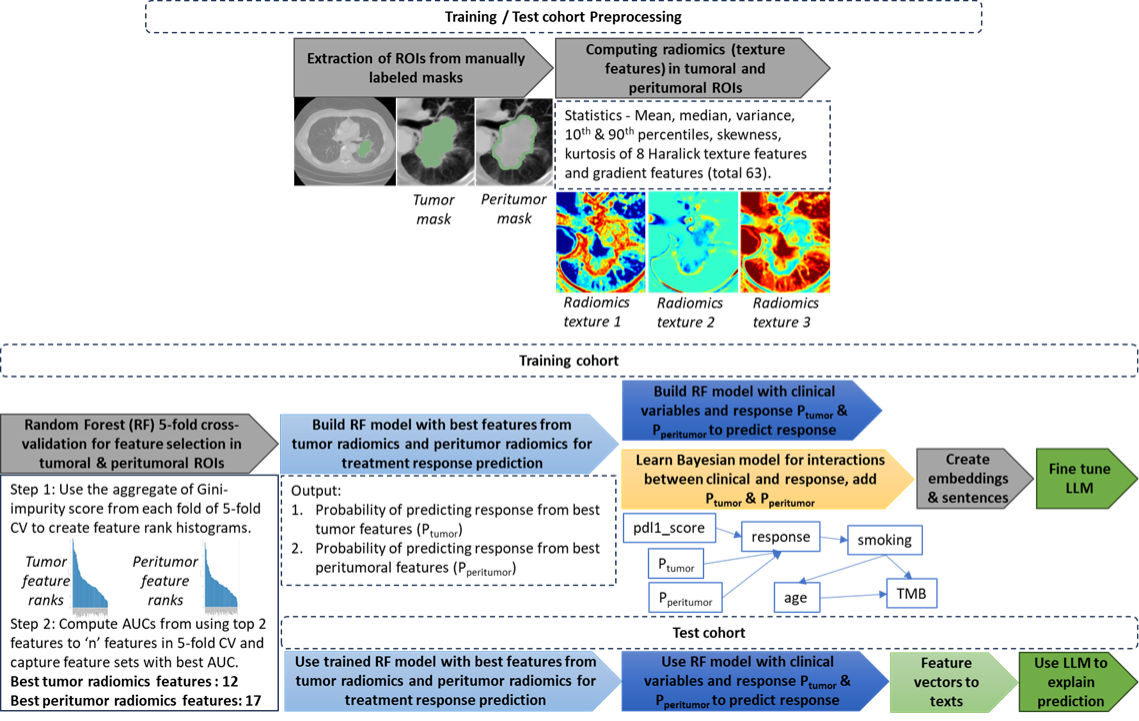
Overview of the study design to predict overall treatment response from CT radiomics and clinical features.

An overview of the study design is shown in Figure 1. Radiomics features were extracted from expert segmented tumor masks from CT volumes. Followed by training a machine learning model to select best tumoral and peritumoral radiomics features; using these top features and clinical variables to train another machine learning model to predict treatment response. The interactions between clinical variables were further learned using a graph structure to create contexts for Large Language Models (LLMs) (GenAI) providing clinically explainable treatment outcomes. The details of each process are provided in the following subheadings.

#### 2.2.1. Feature Extraction from CT

Expert segmented tumor masks were available for all CT volumes. The in-plane x-y resolution was between 0.59 mm–0.98 mm (median = 0.83 mm) and the z-resolution was between 4.8 mm and 5.0 mm (median = 5.0 mm) for all the CT volumes. CT subvolumes were created centering the tumor masks and adding −30, +30 pixels in x-, y-directions and −10, +10 pixels in z-directions were created for radiomics processing. Peritumoral regions were created by dilating the tumor masks 5 × 5 pixels using morphological binary dilation, i.e., median = 4.15 mm × 4.15 mm in each axial (x-y) slice to tackle the anisotropy in dimensions. This peritumoral margin is in concordance with previous research and wider margins do not necessarily provide higher predictive performance of treatment response [29,30]. The tumor mask was then subtracted from the dilated region to create the peritumoral mask. The CT subvolumes were then normalized between 0 and 1 using min-max normalization for extraction of texture features. Three-dimensional Haralick radiomics texture features [31] that leverages Gray-Level Co-Occurrence Matrices (GLCMs) were computed with offset 1 (i.e., within 3 × 3 × 3 neighborhood) using an internal Insight Toolkit (ITK) pipeline. This resulted in 13 sets (13-symmetric GLCMs from 26 neighbors of a voxel) of 8 texture maps such as Energy, Entropy, InverseDifferenceMoment, Inertia, ClusterShade, ClusterProminence, Correlation and HaralickCorrelation, for each voxel. The 13 values were then averaged to one texture map resulting in single maps for each of the 8 Haralick texture features. The gradient features were computed within 3 × 3 × 3 windows using PyRadiomics. Radiomics feature statistics such as mean, median, variance, skewness, kurtosis, 10th and 90th percentile were computed within the masked tumor and peritumor regions, resulting in a set of 63 features for each of the tumor and peritumor regions that were further downselected using feature selection methods in Section 2.2.2.

#### 2.2.2. Feature Selection

A two-stage feature selection method was used to select the best performing tumoral and peritumoral radiomics features using the training dataset (n = 140 subjects). Both stages used Random Forest (RF) [32] as the classifier in a 5-fold cross-validation strategy. In the first-stage, RF was used to predict treatment response, and mean decrease in impurity (MDI) was computed for each fold of cross-validation and averaged across the folds. MDI essentially indicates how much a feature contributes in the model’s ability to separate the data into purer groups by splitting on this feature. Higher MDI signifies greater importance.

Due to the high cardinality of features (n = 63), it was difficult to decide on the top-performing features using the MDI criterion alone. Therefore, a second-stage of feature selection was devised to select the subset of top-performing features. Features from the sorted MDI criterion (decreasing order) were added incrementally to build one RF classifier (predicting treatment response) for each increment. Each classifier was validated using 5-fold cross-validation and the average AUC was recorded for each feature subset that led to selection of the subset with top classification performance.

#### 2.2.3. Model Training and Validation

To understand the effects of peritumoral ($f_{pT}$) and tumoral features ($f_{T}$) separately, we built two separate RF classifiers with the top 12 tumoral features (RF-train-val-$f_{T}$) and 17 peritumoral features (RF-train-val-$f_{pT}$) obtained from Section 2.2.2 to predict the probabilities of treatment responders vs. non-responders, i.e., $p_{T}$ and $p_{pT}$ respectively. Similarly, the two sets of top tumoral and peritumoral features were combined to build a RF classifier (RF-train-val-$f_{T}$-$f_{pT}$) to predict the probability of responders vs. non-responders as $p_{T\_all}$. A 3-fold cross-validation was performed for all these experiments on the training set to obtain prediction probabilities for all training data samples. Additionally, another classifier RF-train-val-Clin-$p_{T}$-$p_{pT}$ that included the clinical variables PD-L1 score, TMB, smoking status and age was trained and validated using 3-fold cross-validation on the training set.

Using the training set, RF classifiers RF-$f_{T}$, RF-$f_{pT}$ and RF-$f_{T}$-$f_{pT}$ were further trained and were validated on the test data, that provided predicted probabilities $p_{T}$, $p_{pT}$ and $p_{T\_all}$ respectively for the test data samples.

The tumoral and peritumoral prediction probabilities $p_{T}$ and $p_{pT}$ of the training data were then used as features along with clinical variables PD-L1 score, TMB, smoking status and age to build the RF prediction model (RF-Clin-$p_{T}$-$p_{pT}$), which was validated only on the test data that included $p_{T}$, $p_{pT}$ in the feature set from the previous stage (RF-$f_{T}$ and RF-$f_{pT}$). As a method of comparison, $p_{T\_all}$ was combined with the clinical variables as above and an RF model (RF-Clin-$p_{T\_all}$) was trained using the training data and validated on test data.

In order to understand the impact of using prediction probabilities of tumoral and peritumoral radiomic feature sets compared to using the features directly, we built another RF classifier (RF-Clin-$f_{T}$-$f_{pT}$) combining clinical variables and the 12 and 17 sets of tumoral and peritumoral features respectively using the training data and this was validated on test data.

#### 2.2.4. Bayesian Network Structure Learning (BNSL)

To understand the interactions between the clinical features and treatment response, Bayesian network structure learning [33] was used on the training cohort. It involves evaluating different network model structures, which are directed acyclic graphs (DAGs) using a search algorithm and a goodness-of-fit score. The search algorithm used was Hill-Climbing Search [34], which is a greedy optimization method that makes iterative local changes to the model structure so that it improves overall score. The score used was BDeu (Bayesian Dirichlet equivalent uniform) [35,36], which is popular choice for discrete Bayesian graphs. The clinical features were all discretized (pdl1 score, TMB and smoking status as per Table 1 and age in bins of 10 years) before optimizing the network structure (BNSL-1). Finally, the discretized probabilities of $p_{T}$ and $p_{pT}$ were added as conditionally dependent variables of treatment outcome. The final Bayesian network model classifier (Bayes-Clin-$p_{T}$-$p_{pT}$-outStruct) was trained on the training cohort and the test cohort was used to to predict outcome.

In order to investigate the effects of including $p_{T}$ and $p_{pT}$ in learning the graph structure, the graph structure learning (BNSL-2) was performed including these along with discretized clinical variables in the Hill-Climbing Search optimization process. This led to a different graph structure; a classifier Bayes-Clin-$p_{T}$-$p_{pT}$-inStruct was further trained on the training data and validated on test data.

It is to be noted that the Bayesian classifiers Bayes-Clin-$p_{T}$-$p_{pT}$-outStruct and Bayes-Clin-$p_{T}$-$p_{pT}$-inStruct were trained to compare their classification performance with RF classifier RF-Clin-$p_{T}$-$p_{pT}$. The learned Bayesian graph structure is a pre-requisite for the LLM fine-tuning in the following section and the trained Bayesian classifier is not used in LLM for natural-language explanation. Therefore, to obtain a simple graph structure for LLM that is clinically more relevant, the probabilities $p_{T}$ and $p_{pT}$ were intentionally left out in BNSL-1 as these are derived variables of radiomics feature-based classifiers from Section 2.2.3 and including these may hinder the optimization process of learning the true associations between clinical variables and outcome.

#### 2.2.5. Bayesian Graph LLM (BgLLM) for Clinically-Explainable Response Prediction (GenAI)

**Figure 2:**
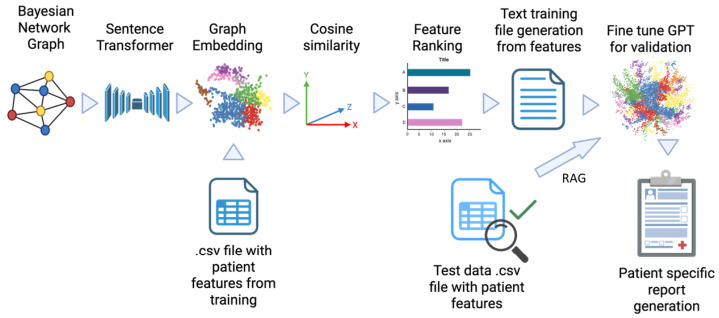
LLM fine-tuning based on sentences generated from Bayesian graph structure and using the fine-tuned LLM (GPT) to explain response prediction in natural language.

Figure 2 shows the workflow of using Bayesian graph learned from BNSL to fine-tuning of GPT for natural language explanations of treatment response predictions. Bayesian network (BN) graphs were created from training set that highlight the primary features driving the outcome for patient populations (Section 2.2.4). The BN with tumor and pertimoral probabilties as conditionally dependent variables of outcome was chosen in this experiment. BNs model relationships between variables at a population level, which can be insufficient for capturing the nuances of individual patient experiences. In order to create a clinically explainable personalized outcome of a patient in natural language involving the top ranking biomarkers, we use the framework of LLM i.e., GPT4 [37]. In a novel framework, we create graph embedding from the BN using a sentence transformer (all-MiniLM-L6-v2) [38]. The embedding aids in querying the graph in natural language or sentences inheriting the node-specific dependencies of the BN. Cosine similarity between the graph embedding vector and features were used to rank the top-ranking variables based on individual patient outcomes in the training dataset. Top-ranking features for all patients in the training dataset and their relation to outcomes were then used in GPT triplet [39] framework to create complete sentences combined with clinical hypotheses and feature/outcome values in the training dataset to create the training file for LLM (See Table 2 for specific clinical hypotheses). This training file was then embedded using OpenAI’s text embedding model, text-embedding-ada-002 [40]. Finally, prompt engineering with Retrieval Augmented Generation (RAG) was used to generate patient-specific clinically explainable reports in natural language for a given patient in the test dataset. Specifically, categorical values of the features along with predicted outcome were used in a query to generate patient-specific clinically explainable report for the patient. Continuous values of the clinical features (TMB, pdl1_score) were converted into categorical high/low based on Table 1, smoking status was considered as positive, average or negative for current, former and never smokers respectively, age was considered high above 65 years, and $p_{T}$ and $p_{pT}$ values were thresholded at 0.7 to indicate high/low probabilities to construct the query.

## 3. Results

### 3.1. Tumoral and Peritumoral Radiomics Texture Features

**Figure 3:**
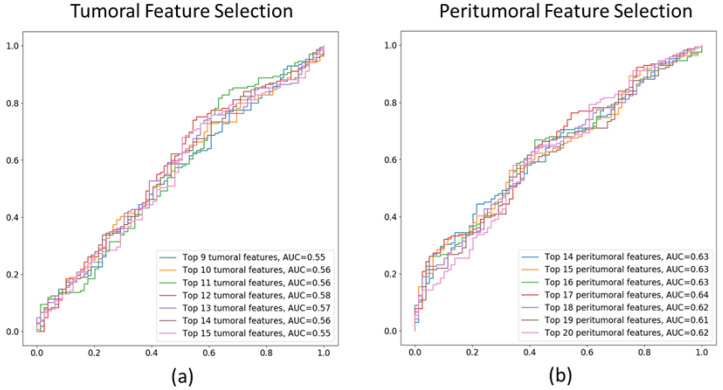
ROC-AUCs of 8–15 top tumoral features and 14–20 peritumoral features are shown in subfigures (**a**) and (**b**) respectively.

In the second-stage of feature selection, AUCs for the RF classifications using incremental sets of top-performing features, which were from the MDI criterion were recorded. Figure 3 shows the ROC and AUCs of the 8–15 top tumoral and 14–20 peritumoral features respectively. The top 12 tumoral radiomics features had the highest AUC = 0.58 and top 17 peritumoral radiomics features had the highest AUC = 0.64. The top 12 tumoral and 17 peritumoral features identified are listed in Table 3.

It was observed that Haralick Correlation, Cluster Shade, Cluster Prominence were important for both tumoral and peritumoral regions, while Entropy and Inverse Difference Moment were important features in the peritumoral region. Higher or lower values of these texture maps may indicate uniformity, assymetry, randomness around the mean intensities in GLCM that are intrinsic measures of tumor heterogeneity with varying densities of cell structures i.e., tumor cells, areas of necrosis, or angiogenesis within and around the tumor [48]. Particularly increased peritumoral heterogeniety that may indicate variation of TILs in the tumor micro-environment that can affect the expression of PD-L1 proteins, and higher TILs is associated with better treatment response to ICIs.

**Figure 4:**
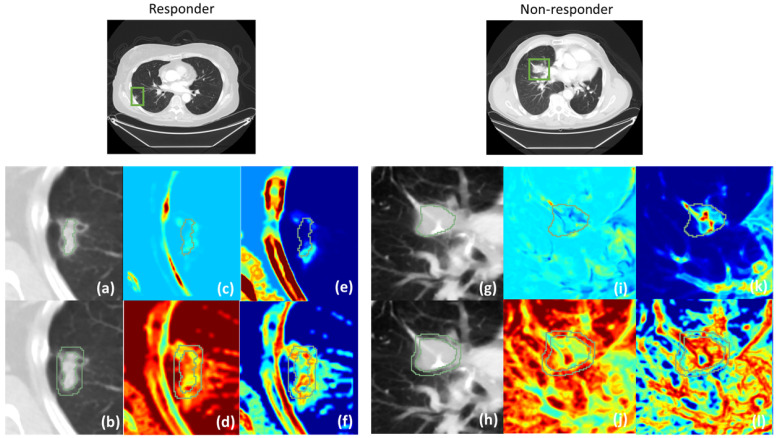
Radiomics texture maps for specific cases of treatment responders and non-responders. The green contours mark the tumoral and peritumoral regions. Each group of subfigures (**a**–**f**) and (**g**–**l**) are cropped regions around the tumor and the radiomics maps of the CT images of the respective responder and non-responder cases in top row. Subfigures (**a**,**b**) and (**g**,**h**) are the cropped CT regions with the tumoral and peritumoral masks overlaid for parenchymal tumors. Subfigures (**c**,**e**) and (**i**,**k**) are Cluster Shade and Haralick Correlation maps for responder and non-responder respectively. Subfigures (**d**,**f**) and (**j**,**l**) are Inverse Difference Moment and Entropy maps respectively for responder and non-responder cases.

Based on the top-performing statistics of the radiomics features we show some of the radiomics texture maps of a responder and a non-responder case in Figure 4. Specifically, Haralick Correlation and Cluster Shade maps are shown with the tumor contour overlays, and Inverse Difference Moment and Entropy radiomics maps are shown with peritumoral delineation overlays. The overlays are shown to indicate that the feature statistics were computed only within the regions of interest.

### 3.2. Performance of ML Models in Predicting Treatment Response

Table 4 shows the Area under the curve (AUC), accuracy and recall values for the ML methods discussed in Section 2.2.3. The method RF-Clin-$p_{T}$-$p_{pT}$ resulted in highest AUC of 0.83 for instance (tumor)-wise (n = 84) response prediction while an AUC of 0.80 was achieved for subject-wise (n = 47) treatment response. The methods RF-train-val-$f_{T}$, RF-train-val-$f_{pT}$ and RF-train-val-$f_{T}$-$f_{pT}$ resulted in values $p_{T}$, $p_{pT}$ and $p_{T\_all}$ respectively for all training data (n = 248 tumors from 140 subjects) and the 3-fold cross-validation results are presented in the table. The predicted values $p_{T}$, $p_{pT}$ and $p_{T\_all}$ of the training dataset were subsequently used to build the ML classifiers from rows 11 through 15. Similarly, the methods RF-$f_{T}$, RF-$f_{pT}$ and RF-$f_{T}$-$f_{pT}$ built using the training dataset, predicted values $p_{T}$, $p_{pT}$ and $p_{T\_all}$ respectively for all test data. The datasets that were used to validate and compute the performance metrics for each method are listed in the table.

### 3.3. Survival Analysis on Test Cohort

**Figure 5:**
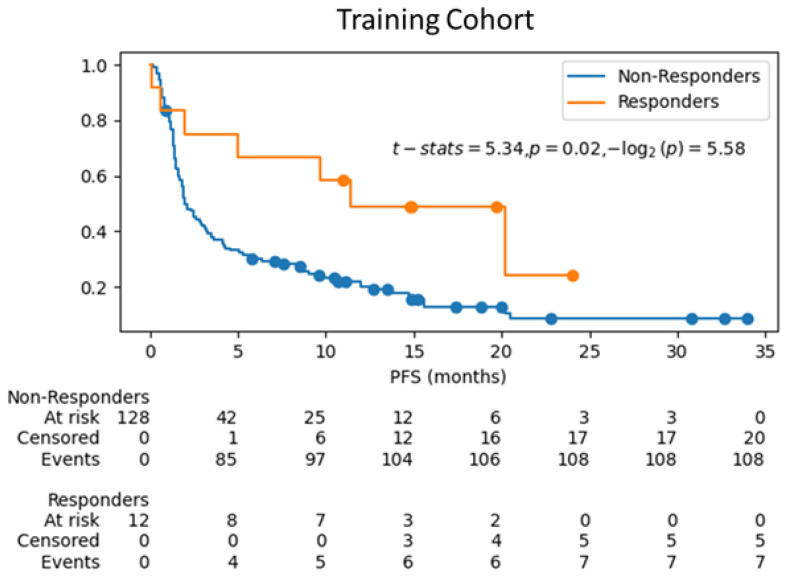
Kaplan–Meier curves for survival analysis of the ML model (RF-train-val-Clin-$p_{T}$-$p_{pT}$) on training cohort using 3-fold cross-validation for prediction of treatment response.

**Figure 6:**
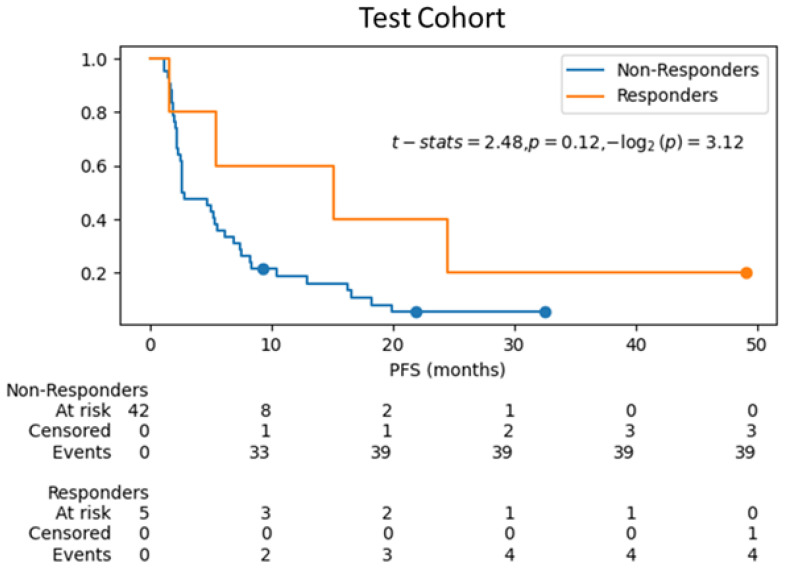
Kaplan–Meier curves for survival analysis of the ML model (RF-Clin-$p_{T}$-$p_{pT}$-Sub) predicting treatment response on test cohort.

Kaplan–Meier curves were estimated for survival analysis between responders and non-responders with the predicted treatment response labels using the ML model RF-train-val-Clin-$p_{T}$-$p_{pT}$-Sub on the training cohort using cross-validation, and for the ML model RF-Clin-$p_{T}$-$p_{pT}$-Sub on test cohort. Figure 5 and Figure 6 show the differences in progression free survival (pfs) between the two groups with censored events for training cohort and test cohort respectively. For the training cohort, the log-rank test shows significant difference (*p*-value < 0.05) in survival between the predicted responders vs. predicted non-responders. Although in the test cohort (Figure 6), the curves visually do not show delayed separation, the log-rank test failed the hypothesis that the survival curves are significantly different, which may be attributed to the sampling difference between the groups in the test cohort (5 predicted responders vs. 42 predicted non-responders) that do not have the power to rule out a real difference and avoid a type two error (false negative) [49].

**Figure 7:**
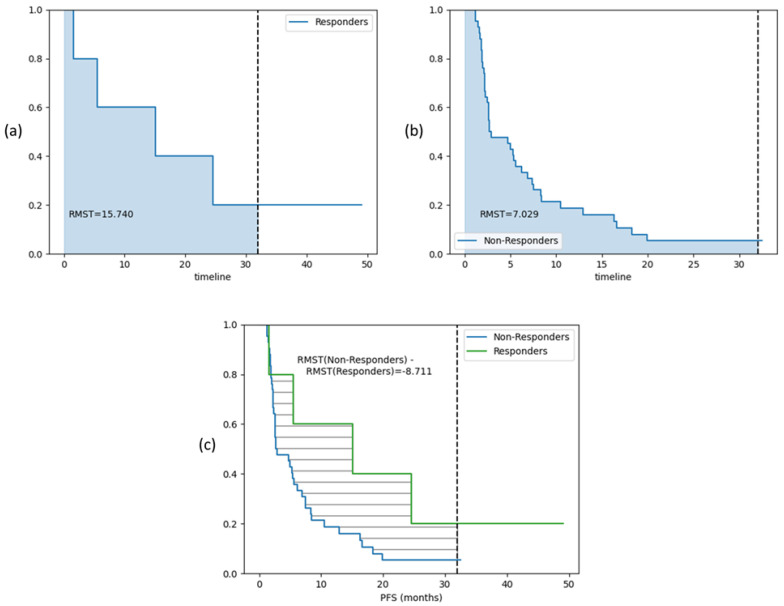
RMST curves showing difference in survival curves from the ML model RF-Clin-$p_{T}$-$p_{pT}$-Sub prediction for treatment response. Subfigures (**a**) and (**b**) show the individual mean survial until 32 months for the responders and non-responders respectively. Subfigure (**c**) shows the difference in mean survival times between the groups, suggesting a gain of 8.7 months in predicting treatment response in test cohort.

Restricted Mean Survival Time (RMST) method can be particularly useful when dealing with low sample sizes in one group, especially in situations where the hazard ratio is not a good measure of treatment effect or when dealing with non-proportional hazards [50]. RMST is defined as the area under the survival curve up to a specific time point. It can be interpreted as the average survival time during a defined time period ranging from time 0 to a specific follow-up time point, which is a straightforward and clinically meaningful way to interpret the contrast in survival between groups. Therefore, RMST may provide valuable information for comparing two survival curves even when the survival difference is not statistically significant between the groups. In this case, RMST was computed from 0 to 32 months and an 8.7 months gain was observed between the mean survivals of non-responders and responders. Figure 7 shows the RMST curves for responders, non-responders and the difference curves for the test cohort.

### 3.4. LLM-Based Clinical Explanation of Treatment Response Prediction

**Figure 8:**
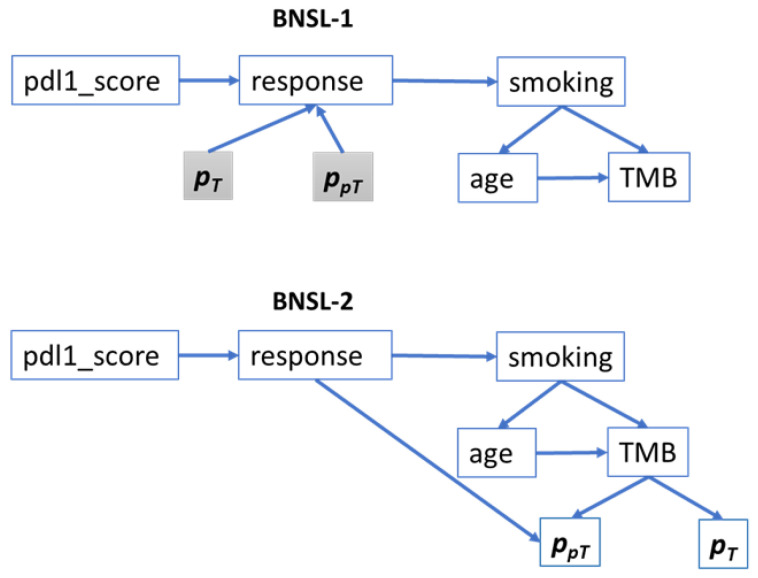
Bayesian graph structures learned from the training dataset for association of clinical variables and response. The prediction probabilities of tumoral and peritumoral radiomics, in shaded boxes were added as dependent variables to the network structure in BNSL-1, while the probabilities were included in graph structure learning in BNSL-2.

The Bayesian graph structures obtained through BNSL-1 and BNSL-2 are shown in Figure 8. The shaded boxes $p_{T}$ and $p_{pT}$ were added as dependent variables of response separately for BNSL-1 i.e., they were not included in structure learning. BNSL-1 was used for LLM due to its simple and clinically relevant network structure.

BNSL-1 in Figure 8 shows that smoking status was incorrectly associated with outcome (directed edge from ‘outcome’ to ‘smoking’). This is not impossible as the Bayesian graph was learned from the training data and a target variable was not specified for graph structure learning. These incorrect connections however, did not impact the creation of training file sentences using sentence transformer as clinically irrelevant sentences were discarded based on the clinical hypotheses.

**Figure 9:**
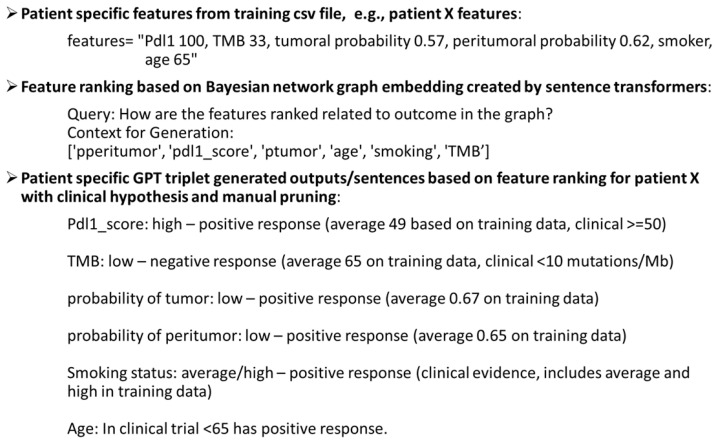
An example of a patient’s features in the training dataset, used to create sentences from graph embedding through feature ranking, combined with clinical hypothesis for the text training file for LLM in Figure 2.

Figure 9 shows the part from graph embedding to sentence generation for LLM text training file, shown in Figure 2 with a patient’s features from the training dataset as an example.

For patient-specific report generation in test dataset, the features were converted to categorical variables for the query. Feature names along with their categorical values and patient outcome were sent to the fine-tuned LLM (GPT4) to generate clinically explainable reports in natural language. Examples are presented in Table 5.

**Figure 10:**
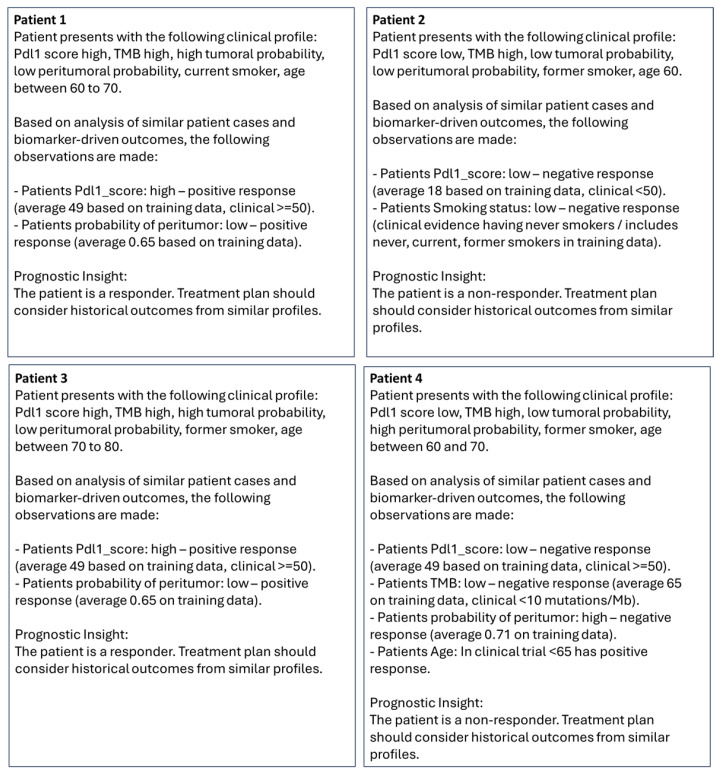
Natural language report generated based on individual patient queries in Table 5.

Specifically, values from individual columns in Table 5 were concatenated to create the query for patients. Reports generated from the queries are shown in Figure 10. Given the features (pdl1_score, TMB, p_tumor, p_peritumor, smoking status, age) and their relationships to treatment response were embedded using the OpenAI embedding model, the LLM ranks top-responses for the queries based on cosine similarities and presents them in natural language along with treatment response to the clinicians for further actions. As observed in Figure 10, for all queries, the top-ranking features (based on high cosine-similarity of the query and generated vectors) and their associated hypotheses were generated by LLM. For Patient 4, we observed irrelevant association with the clinical hypothesis (in parenthesis) of pdl1-score high with a non-responder although this subject had zero pdl1-score.

**Figure 11:**
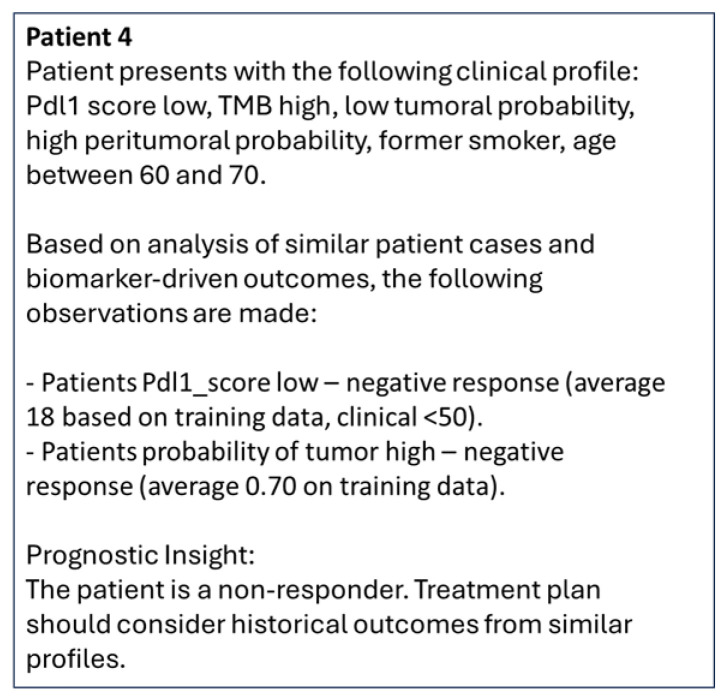
Natural language report re-generated with higher AR difference threshold (τ = 0.05) in LLM for Patient 4 in Table 5.

To mitigate this issue, a threshold τ measuring the difference of Answer Relevancy (AR) i.e., the difference in cosine similarities between embeddings of the set of generated responses $E_{g}$ to the embedding of the original question $E_{o}$ was used to generate more relevant responses. Lower threshold (τ = 0.01) led to more generated responses with chances of irrelevance, while higher threshold (τ = 0.05) led to fewer and relevant generated responses by the LLM as shown in Figure 11 for Patient 4.

In order to measure the relevance of the generated LLM reports, we further computed the mean Answer Relevancy Metric (ARM) i.e., mean the cosine similarity of the generated sentences for all test queries (see Equation (1) [51]). Where, $E_{g}$ is the embedding of the generated question *i*, $E_{o}$ is the embedding of the original question, and *N* is the number of generated responses. ARM outputs a score between 0.0 and 1.0, assessing the consistency of the generated answer based on the reference ground truth answers. Values closer to 1.0 signify higher relevance. ARM for Patient 4 reports in Figure 10 and Figure 11 correspond to ARM values 0.83 ± 0.02 and 0.85 ± 0.07 with thresholds (τ) 0.01 and 0.05 respectively. Table 6 shows the ARM mean, standard deviation of all test subjects with modified difference in relevancy thresholds (τ) for LLM generation. It is to be noted, the queries were well-structured in this study, hence the generated reports do not suffer significantly from randomness in responses.

(1) $$ARM=\frac{1}{N}\sum_{i=1}^{N}\cos(E_{g},E_{o})=\frac{1}{N}\sum_{i=1}^{N}\frac{E_{g}\;\cdot \;E_{o}}{∥E_{g}∥∥E_{o}∥}$$

## 4. Discussions

While ICIs have extensive application prospects for advanced NSCLC, their emerging resistance to ICIs necessitate the therapeutic diversification. Therefore, developing effective AI/ML methods to integrate multimodal, multiomics datasets can guide the selection of personalized therapeutic approaches for individual patients, particularly by enabling earlier identification of non-responders and refining clinical decision-making to improve NSCLC survival.

In this work, we explored the use of radiomics and clinical variables in predicting treatment response. Tumoral and peritumoral radiomics texture features were extracted with a two-step feature selection process, then combined with clinical features to predict response to immunotherapy for NSCLC. Finally, a clinically-explainable Bayesian graph-LLM-based technique was used to summarize the prediction in natural language.

### 4.1. Comparison with Prior Studies

Overall, we observed RF classification models had improved accuracy than Bayesian classification as RF models are efficient in handling non-linear relationships between features and target variable [52], while Bayesian classifiers assume feature independence. Among RF models in Table 4, we noticed that use of peritumoral radiomics features show improved accuracy compared to using radiomics features within the tumor region in both training-validation and test cohorts. Particularly, the performance of treatment response is compromised when tumor region radiomics is combined with peritumoral radiomics. This is an interesting drift from the recent research by Huang et al. [30] and Wang et al. [53] where the combination of tumoral and peritumoral radiomics have been shown to improve performance and also tumoral radiomics features were more predictive than peritumoral radiomics [53]. However, the research of Wu et al. [54] and Huang et al. [30] corroborate our findings that peritumoral radiomics drive the prediction of response to immunotherapy or chemotherapy in NSCLC. This phenomenon can be explained biologically, as T-cell infilitration, antigen presentation and immune exclusion occur in the peripheral zone, making it more reflective of immune responsiveness. In addition, this area is often more dynamic unlike the tumoral region that may be hypoxic, necrotic or fibrotic.

Multimodal integration of pre-treatment CT radiomics, PDL1-IHC radiomics features with genomics and clinical variables was shown in Vanguri et al. [28] and Peng et al. [55] using the dataset in [28]. In both works, tumoral radiomics were computed and peritumoral radiomics features were not considered. Specifically, in the work by Vanguri et al. [28], radiomics texture features of tumoral regions were not explored and only PDL1-IHC radiomics texture features were considered in the analysis. The analysis by Peng et al. [55] however, was robust as the training and test cohorts were two separate public datasets compared to 5-fold cross-validation with all data in Vanguri et al. [28], which was due to the unavailability of multimodal data for every subject. Despite the data availability constraint we attempted to divide the dataset [28] into separate training and test cohorts as we only considered CT and clinical variables in our multimodal analysis. One major difference between our work and Vanguri et al. [28] is the dataset split for training/validation and testing, which were identified randomly using k-stratified sampling in our case to maintain uniform distribution of responder vs. non-responder subjects across each subset.

Although clinical features such as PDL1 score, TMB, and other clinical variables listed in Table 1 were considered in the analysis of Vanguri et al. [28], the combination of more than one clinical variable was not studied. It has been shown by Castellanos et al. [56] and Kao et al. [57] that a combination of PDL1 score, TMB and/or dNLR (derived neutrophil-to-lymphocyte ratio) is a better predictor of immunotherapy response compared to individual clinical variables. Similarly other clinical factors such as smoking status, age, gender may also play a role. In this work, we show that a combination of PDL1-score, TMB, smoking status and age is a better predictor of immunotherapy response than tumoral or peritumoral radiomics alone or their combinations (Table 4). Interestingly, the combination of radiomics features with clinical variables (RF-Clin-$p_{T}$-$p_{pT}$) led to a modest improvement of prediction power compared to the one with clinical variables only (RF-Clin). This observation is in concordance with a study of immunotherapy response where addition of radiomics features with clinical variables did not increase the prediction power significantly [58,59,60].

### 4.2. Interpretability

An interesting observation of our work is the improvement in prediction by using the probabilities of treatment response, both from tumoral and peritumoral radiomics as features (RF-Clin-$p_{T}$-$p_{pT}$) compared to using the radiomics features directly in a model (RF-Clin-$f_{T}$-$f_{pT}$). We think this is due to curse of dimensionality of the features, which was not mitigated even after using dimensionality reduction techniques such as PCA. We think this manner of decoupling radiomic features and clinical variables leads to straightforward interpretation where clinicians may be left to interpret the impact (high/low) of peritumoral or tumoral radiomic features in predicting response instead of the complicated feature values. While the important radiomics texture maps will be at disposal for detailed analysis.

Creation of knowledge graph from Graph-RAG, starting from large volumes of text may not be perfect as the graph structure may exhibit incorrect feature dependencies with clinical outcomes. Moreover, insertion of new derived features and biomarkers (like tumoral and peritumoral response probabilities) that can predict outcome is not possible. In order to mitigate the above issues we focused on using a feature set of clinical variables and derived biomarkers to correctly predict treatment response using the RF framework, followed by BNSL to learn an accurate graph structure from these features and adding the response probabilities of tumoral and peritumoral regions as dependent features to treatment response. Although BNs provide population-specific knowledge, graph embeddings from BN allowed creation of sentences from patient-specific feature rankings that were used to fine-tune the LLM. The clinical hypotheses (in Table 2) used to generate the training dataset for LLM involved unimodal associations between the clinical variables and treatment outcome based on literature, while in reality much more complex associations exist and including these is in the scope of our future study to generate more complex reports. Most importantly, our work aimed to interpret the predictions in terms of variables that are comprehensible by clinicians, including response probabilities driven by tumoral and peritumoral radiomics instead of presenting raw radiomics values to clinicians and despite the tacit role of radiomics in prediction.

### 4.3. Limitations

Our study has several limitations. Firstly, we do not leverage all modalities available in the MSKCC data [28] predominantly due to sparsity of modalities across subjects. Therefore, we are limited in sample size for improved accuracy with all multimodal data. The top performance of our method (RF-Clin-$p_{T}$-$p_{pT}$-Sub) combining radiomics and clinical variables only was similar to Vanguri et al. [28] combining radiomics from CT, PDL1-IHC slides, genomics and clinical variables (AUC = 0.80) although in a smaller and separate test cohort of MSKCC compared to a 5-fold cross validation used in Vanguri et al. [28]. However, the performance of RF-$p_{T}$-$p_{pT}$ using radiomics features alone was inferior. In fact, the modest performance of radiomics texture features alone was also elucidated in the feature selection strategy with AUCs in Figure 3, which was cross-validation on training cohort. The shape or structural radiomics for the tumoral regions and a broad-range of radiomics texture features (wavelet, etc.,) were not explored in this study, the inclusion of which may lead to further improvement in prediction of treatment response using only CT radiomics-based features.

Our study was on retrospective data, and from a single institution and the sample size was modest. Therefore, despite having separate training and testing cohorts we think our ML model has some bias and therefore may not be generalizable for multicenter data with diverse clinical settings, such as demographics, treatment protocols that are different across institutions.

### 4.4. Future Directions

An area of future research could be using serial imaging (PET/CT) for biomarker analysis, since pre-treatment images are likely reflective of initial responses to immunotherapy. Secondary resistance to immunotherapy will likely not be captured on the initial scans. In such cases, serial radiomic biomarkers and use of multiomics datasets combined with AI/ML methods can guide selection of personalized therapeutic approaches for patients by earlier detection of acquired secondary resistance, and help refine clinical decision improving survival in NSCLC.

## Figures and Tables

**Table 1:** Characteristics of patients in the training and test cohort.

Characteristics	Training Cohort (n = 140 Patients (%)) (n = 248 Lesions (%))	Test Cohort (n = 47 Patients (%)) (n = 84 Lesions (%))
*Sex*		
Male	62 (44%)	25 (53%)
Female	78 (56%)	22 (47%)
*Age in yrs*		
Median	68	67
Minimum	38	38
Maximum	93	83
*Smoking Status*		
Current	91 (65%)	29 (62%)
Former	33 (24%)	11 (23%)
Never	16 (11%)	7 (15%)
*PD-L1 Score (%)*		
0	71 (51%)	17 (36%)
1–49	25 (18%)	9 (19%)
≥50	44 (31%)	21 (45%)
*TMB*		
≥10 mutations/Mb	139 (99%)	47 (100%)
<10 mutations/Mb	1 (1%)	0 (0%)
*Overall Treatment Response*		
Non-responders	102 (73%)	34 (72%)
Responders	38 (27%)	13 (28%)
*Lesion Type* ^1^		
Parenchymal	152 (61%)	51 (61%)
Pleural	21 (9%)	11 (13%)
Lymph node	75 (30%)	22 (26%)

^1^ For ‘Lesion Type’, the percentages are based on total no. of lesions. Other percentages are based on total no. of patients.

**Table 2:** Clinical hypotheses for NSCLC immunotherapy response with features values and associated outcomes in training dataset for LLM training.

Pdl1_score: high—positive response (average 49 based on training data, clinical ≥ 50 [41])
Pdl1_score: low—negative response (average 18 based on training data, clinical < 50 [41]
TMB: high—positive response (average 74 on training data, clinical > 10 mutations/Mb [42,43,44])
TMB: low—negative response (average 65 on training data, clinical < 10 mutations/Mb [42,43,44])
p_tumor: high—negative response (average 0.70 on training data)
p_tumor: low—positive response (average 0.67 on training data)
p_peritumor: high—negative response (average 0.71 on training data)
p_peritumor: low—positive response (average 0.65 on training data)
Smoking status: former/current—positive response (clinical evidence with former and current smokers [45,46], includes former and current smokers in training data))
Smoking status: never smoker—negative response (clinical evidence with never smokers [45,46]/includes never, current, former smoker in training data)
Age: Average same in training data between responder and non-responder (mean 67), median slightly lower at 67 in responders than non-responders at 68 but <65 has positive response than ≥65 in some clinical evidence [47].

**Table 3:** Tumoral and peritumoral radiomics feature list after feature selection.

Tumoral Features	Peritumoral Features
Gradient_10thPercentile	Entropy_variance
Gradient_median	Correlation_median
Correlation_skewness	InverseDifferenceMoment_variance
Correlation_variance	ClusterShade_mean
ClusterShade_mean	Energy_variance
Gradient_mean	InverseDifferenceMoment_kurtosis
ClusterShade_skewness	HaralickCorrelation_skewness
ClusterProminence_90thPercentile	Entropy_kurtosis
Gradient_90thPercentile	Entropy_90thPercentile
HaralickCorrelation_skewness	Correlation_mean
HaralickCorrelation_variance	Gradient_90thPercentile
ClusterShade_variance	HaralickCorrelation_mean
	ClusterProminence_mean
	Gradient_10thPercentile
	Entropy_median
	HaralickCorrelation_10thPercentile
	ClusterProminence_variance

**Table 4:** Performance metrics for the ML methods and variations to predict treatment response. The third column in this table shows the dataset that was used to validate and measure the performance for each of the listed methods in second column. The methods with ‘train-val’ in names are results on training dataset (n = 248 tumors from 140 subjects) to derive values $p_{T}$, $p_{pT}$ and $p_{T\_all}$ on all training data. All other methods were evaluated on the test cohort of n = 84 tumors from 47 subjects. The methods with ‘Clin’ in names comprise of clinical variables pdl1 score, TMB, smoking status and age. Performance metrics are reported for instance (tumor)-wise classification except methods RF-train-val-Clin-$p_{T}$-$p_{pT}$-Sub & RF-Clin-$p_{T}$-$p_{pT}$-Sub shows results for subject-wise response prediction. $f_{T}$, $f_{pT}$ are top tumoral and peritumoral radiomics features, $p_{T}$, $p_{pT}$, and $p_{T\_all}$ are treatment response probabilities using $f_{T}$, $f_{pT}$ and their combination respectively. The suffixes ‘-inStruct’ and ‘-outStruct’ in the method name (1st column) represent that probabilities $p_{T}$ and $p_{pT}$ were considered and not considered respectively in the BNSL before the Bayesian classifiers were trained.

	Method	Validation	AUC	Accuracy	Recall
1	RF-train-val-$f_{T}$	Training	0.58	0.66	0.95
2	RF-train-val-$f_{pT}$	Training	0.64	0.69	0.95
3	RF-train-val-$f_{T}$-$f_{pT}$	Training	0.60	0.66	0.95
4	RF-train-val-Clin-$p_{T}$-$p_{pT}$	Training	0.73	0.72	0.94
5	RF-train-val-Clin-$p_{T}$-$p_{pT}$-Sub	Training	0.72	0.76	0.96
6	RF-$f_{T}$	Test	0.60	0.73	1.00
7	RF-$f_{pT}$	Test	0.62	0.75	1.00
8	RF-Clin	Test	0.81	0.79	0.90
9	RF-$f_{T}$-$f_{pT}$	Test	0.58	0.73	1.00
10	RF-Clin-$f_{T}$-$f_{pT}$	Test	0.67	0.75	0.98
11	RF-Clin-$p_{T\_all}$	Test	0.83	0.79	0.90
12	RF-Clin-$p_{T}$-$p_{pT}$	Test	0.83	0.80	0.95
13	RF-Clin-$p_{T}$-$p_{pT}$-Sub	Test	0.80	0.74	0.94
14	Bayes-Clin-$p_{T}$-$p_{pT}$-outStruct	Test	0.70	0.73	0.77
15	Bayes-Clin-$p_{T}$-$p_{pT}$-inStruct	Test	0.70	0.73	0.77

**Table 5:** Examples of patient-specific queries into fine-tuned LLM. Features are converted to categorical variables (in columns) and concatenated for query (in rows).

Patient 1	responder	pdl1_score high	TMB high	high tumoral probability	low peritumoral probability	current smoker	age between 60 and 70
Patient 2	non responder	pdl1_score low	TMB high	low tumoral probability	low peritumoral probability	former smoker	age 60
Patient 3	responder	pdl1_score high	TMB high	high tumoral probability	low peritumoral probability	former smoker	age between 70 and 80
Patient 4	non responder	pdl1_score low	TMB high	low tumoral probability	high peritumoral probability	former smoker	age between 60 and 70

**Table 6:** Answer Relevancy Metric (ARM) for generated responses based on the queries before and after modification of τ threshold in LLM for all test subjects.

Subjects	ARM (τ = 0.01)	ARM (τ = 0.05)
Test subjects (n = 47)	0.84 ± 0.02	0.86 ± 0.04

## Data Availability

MSKCC study data available publicly at https://www.synapse.org/#!Synapse:syn26642505, accessed on 12 August 2025.
